# Gene expression assay and Watson for Oncology for optimization of treatment in ER-positive, HER2-negative breast cancer

**DOI:** 10.1371/journal.pone.0200100

**Published:** 2018-07-06

**Authors:** Yun Yeong Kim, Se Jeong Oh, Yong Soon Chun, Woon Kee Lee, Heung Kyu Park

**Affiliations:** 1 Department of Surgery, Breast Cancer Center, Gil Medical Center, Gachon University College of Medicine, Incheon, Republic of Korea; 2 Department of Surgery, Breast Cancer Center, Catholic University Saint Mary’s Hospital, Incheon, Republic of Korea; 3 Department of Surgery, Gil Medical Center, Gachon University College of Medicine, Incheon, Republic of Korea; University of North Carolina at Chapel Hill School of Medicine, UNITED STATES

## Abstract

**Background:**

Personalized treatment for cancer patients is a hot topic of debate, particularly the decision to initiate chemotherapy in patients with Estrogen receptor (ER)-positive, HER2-negative tumors in the early stages of breast cancer (BC). Owing to significant advancements in information technology (IT) and genomics, clinicians are increasingly attaining therapeutic goals rapidly and safely by effectively differentiating patient subsets that require chemotherapy. IBM Watson for Oncology (WFO) is a cognitive computing system employed by clinicians to provide evidence-based treatment options for cancer. WFO aids in clinical diagnosis, with claims that it may be superior in performance to human clinicians. The current study was based on the hypothesis that WFO alone cannot effectively determine whether or not chemotherapy is essential for the subset of ER-positive, HER2-negative BC patients.

**Patients and methods:**

From December 2015 to July 2017, 95 patients with ER-positive, HER2- negative BC subjected to treatment were retrospectively examined using WFO, and outputs compared to real clinical practice. Treatment options were suggested by WFO, and WFO recommendations calculated both with and without data from the gene expression assay (GEA).

**Results:**

WFO without GEA was unable to determine the groups of patients that did not require chemotherapy. Concordant therapeutic recommendations between real clinical practice and WFO without GEA were obtained for 23.2% of the patient group. On the other hand, the results of WFO with GEA showed good clinical applicability. Sensitivity, specificity, positive predictive and negative predictive values of WFO with GEA were 100%, 80%, 61% and 100%, respectively.

**Conclusions:**

Our collective findings indicate that WFO without the gene expression assay has limited clinical utility.

## Introduction

Biomarkers in breast cancer (BC) are essential contributors to therapeutic assessment. In addition to conventional prognostic factors, such as tumor size, grade and nodal status, three established predictive biomarkers, estrogen (ER) and progesterone (PR) receptors and human epidermal growth factor receptor 2 (HER2) status, are utilized for making treatment decisions. Substantial evidence suggests that patients with the ER-positive, HER2-negative subtype in early-stage BC carry a much better prognosis [1–4]. ER-positive, HER2-negative breast cancers are frequently treated with adjuvant chemotherapy, although most show favorable outcomes with endocrine therapy alone [5]. The advantages of chemotherapy in addition to regular hormonal therapy for hormone receptor-positive early BC remains a subject of debate [6, 7]. Calculation of individual response predictions remains poor without inclusion of the gene expression dataset [1, 8, 9]. Several tests in recent years have assessed the expression profiles of cancer-related genes to provide prognostic information on disease-free and overall survival [10, 11]. Oncotype Dx is a RT-qPCR-based 21-gene assay using RNA from formalin-fixed paraffin-embedded (FFPE) tissue, which is composed of 16 cancer genes primarily related to tumor proliferation [10, 12]. The Oncotype Dx recurrence score (RS) is widely used for clinical purposes. Other prognostic scores to estimate residual risk in endocrine-treated patients include the PAM50 risk or recurrence (ROR) score [13], Breast Cancer Index (BCI) [14], and IHC4 test [15]. Oncotype Dx was initially analyzed in clinical trials in 2004 and shown to be capable of quantifying the probability of distant recurrence and likelihood of response to chemotherapy in early hormone receptor-positive BC [4, 10]. The utility of RS was validated in the NSABP B-14 trial in 2004 on 645 patients. A subsequent NSABP B-20 trial in 2006 involving 651 patients validated the benefits of additional chemotherapy in patients with high RS. On the other hand, patients with low RS did not benefit from additional chemotherapy [4, 16].

**Fig 1:**
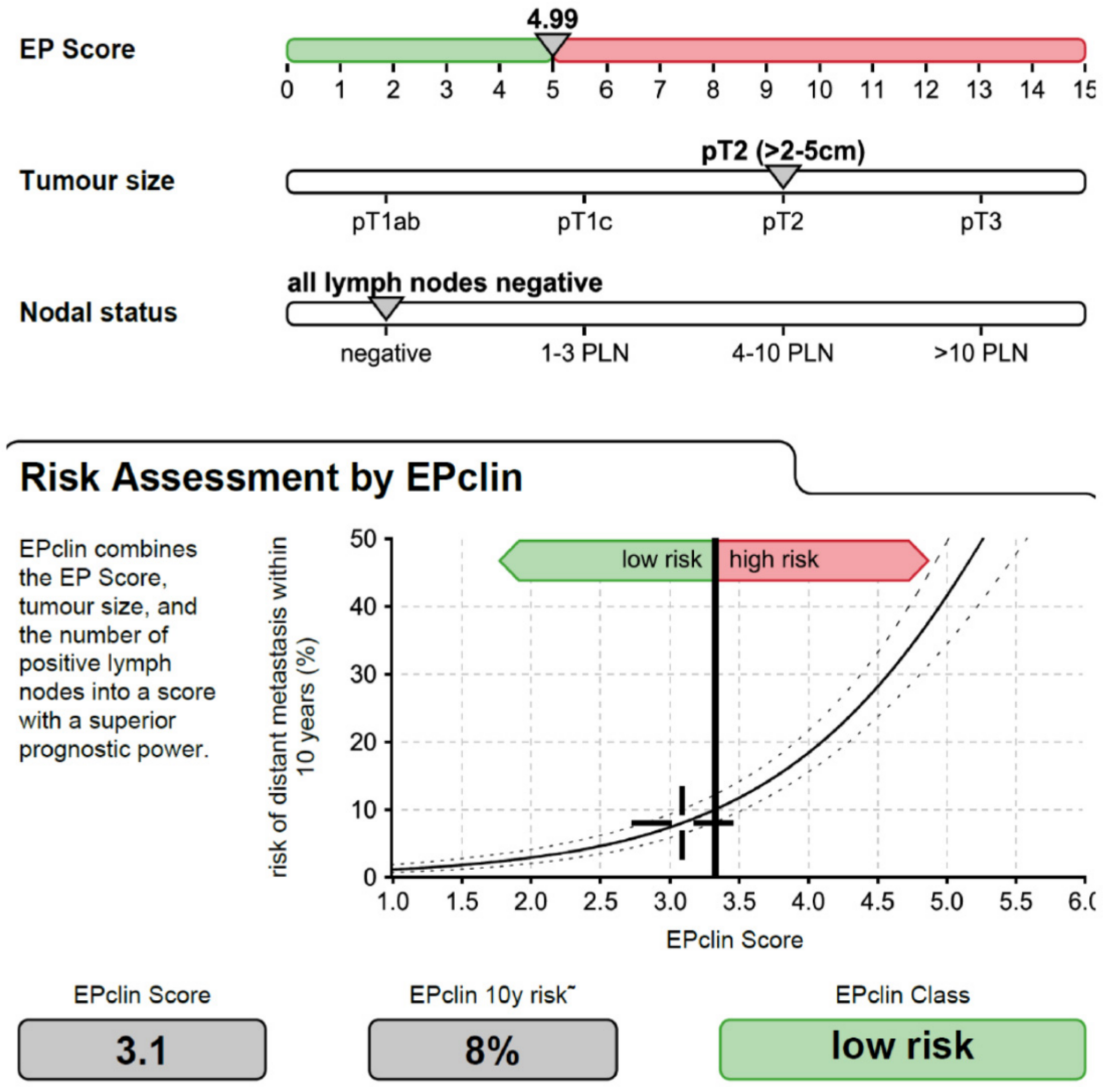
Patient reports based on the EndoPredict (EP) assay. This patient is premenopausal woman with T2N0M0, luminal A subtype breast cancer. She was in low risk EPclin class, indicative of a low 10-year risk of distant metastasis. EP assay is clinically useful to choose adjuvant chemotherapy.

The EndoPredict (EP) assay is an RT-qPCR-based 12-gene test based on RNA from FFPE tissue in hormone receptor-positive, HER2-negative, nodal-negative and -positive BC managed with adjuvant hormonal treatment alone [17] that has been designed to integrate genomic and clinical information. The EP score generated provides significant prognostic information in addition to conventional prognostic clinicopathologic parameters such as tumor size and nodal status. Combination of the molecular EP score with clinicopathologic risk factors results in an EPclin score that facilitates the identification of risk groups with significant differences in 10-year distant recurrence rates. The molecular clinicopathologic EPclin score outperformed established prognostic parameters in two patient cohorts, ABCSG-6 and ABCSG-8 [17–19]. EPclin low-risk patients had a 10-year risk of distant recurrence of 4% and were adequately treated with adjuvant endocrine therapy only. In contrast, for EPclin high-risk patients with 10-year distant recurrence risk of 28% (ABCSG-6) and 22% (ABCSG-8), endocrine therapy alone was not considered sufficient and additional adjuvant treatment indicated [17]. Fig 1 depicts an easy-to-perform multi-gene tool in clinical practice. The EPclin score has strong potential to assist in optimizing adjuvant therapy by reducing the risk of both under- and overtreatment.

Recent advances in information technology (IT) have included the development of IBM Watson for Oncology (WFO), a Memorial Sloan Kettering Cancer Center-trained cognitive computing system, to provide clinicians with evidence-based treatment options for cancer [20–22]. When an oncologist inputs a clinical question into the system, WFO generates a list of hypotheses in response. WFO treatment options are presented in three categories: ‘Recommended’, ‘For consideration’, and ‘Not Recommended’ [23]. Therapeutic options suggested by WFO are easy to access in the clinic, and both ‘Recommended’ and ‘For consideration’ categories selected for treatment. WFO can be compared to a car navigation system that helps clinicians reach therapeutic goals rapidly and safely. This computerized system may be helpful in guiding personalized treatment and its potential superiority to human clinicians in terms of performance is an issue under debate. Using molecular clinicopathologic data, human clinicians are able to determine whether or not a particular subset of patients with ER-positive HER2-negative BC would benefit from chemotherapy. With the significant advancements in genomics, available clinical data can be effectively utilized to provide discriminatory evidence that supports informed therapeutic decision-making.

This retrospective study was based on the hypothesis that the current WFO guidelines do not provide sufficient information for clinicians to make informed decisions on personalized therapy. Clinicopathologic data with or without genomic data were entered into the WFO formula for each patient in a retrospective manner. Therapeutic options generated by WFO were compared with those recommended in real clinical practice for each patient of the study group.

## Methods

### Study population

Our study included 95 patients treated for early BC in Gachon University Gil Medical Center between Dec 2015 and July 2017. The study group was limited to estrogen receptor (ER)-positive and HER2-negative breast cancer (BC) for which EP assays were conducted successfully. All patients underwent breast conserving surgery or total mastectomy as well as sentinel lymph node biopsy in cases of clinically and radiologically negative axillary lymph nodes. Level I and II axillary dissection were performed for macrometastases or micrometastases of the sentinel lymph node. Formalin-fixed paraffin-embedded (FFPE) tissue samples were derived from patients with primary ER-positive, HER2-negative BC. All FFPE tumor blocks were collected at the time of surgery prior to adjuvant therapy.

Clinicopathologic data (tumor size, nodal status, grading and Ki-67 level) were extracted from the pathologic reports. The Ki-67 cutoff point was applied based on the St. Gallen guidelines [24].

### Ethical statement

The protocol for this retrospective study was reviewed and approved by the Institutional Review Board (approval number: GBIRB2017-329) of the Gachon University Gil Medical Center. As the study was conducted on a total of 95 consecutive patients from our database and involved no more than minimal risk for the subjects, the Institutional Review Board approved our request for waiver of informed consent. Recommendations of the Declaration of Helsinki for biomedical research engaging human subjects were also followed.

### RNA extraction and assessment of EndoPredict

EP, a new RNA-based multigene scoring system predicting the likelihood of distant recurrence in patients with early stage, ER-positive and HER2-negative BC treated with adjuvant endocrine therapy, has been described in the literature [17]. EP scores, in combination with the clinical risk factors tumor size and nodal status (EPclin), were generated for all tumor samples based on assessment of eight genes of interest implicated in carcinogenesis (GOI, genes of interest: BIRC5, UBE2C, DHCR7, RBBP8, IL6ST, AZGP1, MGP and STC2) and three normalization genes (CALM2, OAZ1 and RPL37A). Total RNA was extracted from FFPE tissue sections using a silica-based, fully automated isolation method (Tissue Preparation System, Siemens Healthcare Diagnostics, VERSANT Tissue Preparations Reagents, Tarrytown, NY, USA) as published earlier [17]. All samples were subjected to quantitative one-step SuperScript III PLATINUM reverse transcriptase PCR (qRT-PCR) with ROX (Invitrogen, Grand Island, NY, USA) on an ABI PRISM 7900HT system (Applied Biosystems, Foster City, CA, USA). Normalized expression levels of GOI as well as EP and EPclin scores were calculated. Finally, samples were classified as low or high risk of distant metastasis according to predefined cutoff values of 5 (molecular risk score, EP) and 3.3 (integrated molecular and clinical risk score, EPclin) [19].

### Watson for oncology

We retrospectively investigated 95 patients treated with or without chemotherapy in a blinded manner using WFO. Following entry of clinicopathologic data into WFO, EPclin results for each patient were additionally entered into ‘Genetic risk assessment’ within WFO. Treatment options recommended by WFO were calculated either in conjunction with or without EPclin results. Currently, the IBM formula uses Oncotype Dx data that facilitate categorization into three groups: low, intermediate and high risk. In contrast, EP score leads to low and high dichotomous classifications. We further examined the concordance in prognostic ability between Oncotype Dx and EP and substituted the EP score for Oncotype Dx. Based on the assessments, possible therapies were assigned into green, yellow, and red “buckets”, specifically, green for recommended, yellow for under-consideration, and red for not-recommended. Therapeutic option in the green bucket were regarded as a single therapeutic approach by WFO. Within this study group, all patients were categorized as suitable for either endocrine therapy alone or with added chemotherapy. Therapeutic option assigned to the yellow “bucket” were not considered in this study.

### Data analysis

Statistical analysis was conducted using SPSS Statistics Version 19.0 (IBM, Armonk, USA). The correlations between EP score and tumor grade and trends between EP score and Ki-67 data were analyzed using the Jonckheere-Terpstra test. Associations between WFO options and real clinical practice were calculated using the chi-square test. We used the logistic regression model to compute the odds ratio for chemotherapy. Graphics were generated with GraphPad Prism 6 (GraphPad Software, Inc., La Jolla, CA, USA).

## Results

### Patients and tumor characteristics

Patients and tumor characteristics are detailed in Table 1. Mean age was 49.9 years (range, 29–75 years) and mean tumor size was 1.97 cm (range, 0.7–5.5 cm). Among the 95 patients under study, 83 (87.4%) were diagnosed with node-negative disease and 12 (12.6%) with node-positive disease.

**Table 1:** Study population characteristics at baseline.

	Overall *N = 95*	Node-negative *N = 83*	Node-positive *N = 12*
**Age, years[mean(SD),range]**	49.9(9.0), 29–75	49.8(8.8),29–75	51(10.6),36–71
**Tumor grade** **Gr1[n(%)]** **Gr2[(n(%))** **Gr3[n(%)]**	10(10.5%) 69(72.6%) 16(16.8%)	7(8.4%) 61(73.5%) 15(18.1%)	3(25.0%) 8(66.7%) 1(8.3%)
**Tumor size, cm[mean(SD),range]**	1.97(0.95),0.7–5.5	1.96(0.98), 0.7–5.5	2.03(0.75). 0.8–3.5
**EP**^a^ **score[mean(SD),range]**	5.67(2.69), 0–12.4	5.76(2.74), 0–12.4	5.05(2.39), 1.9–9.3

^a^EndoPredict assay for tumor genomic information.

### Test performance and distribution of risk groups

**Fig 2:**
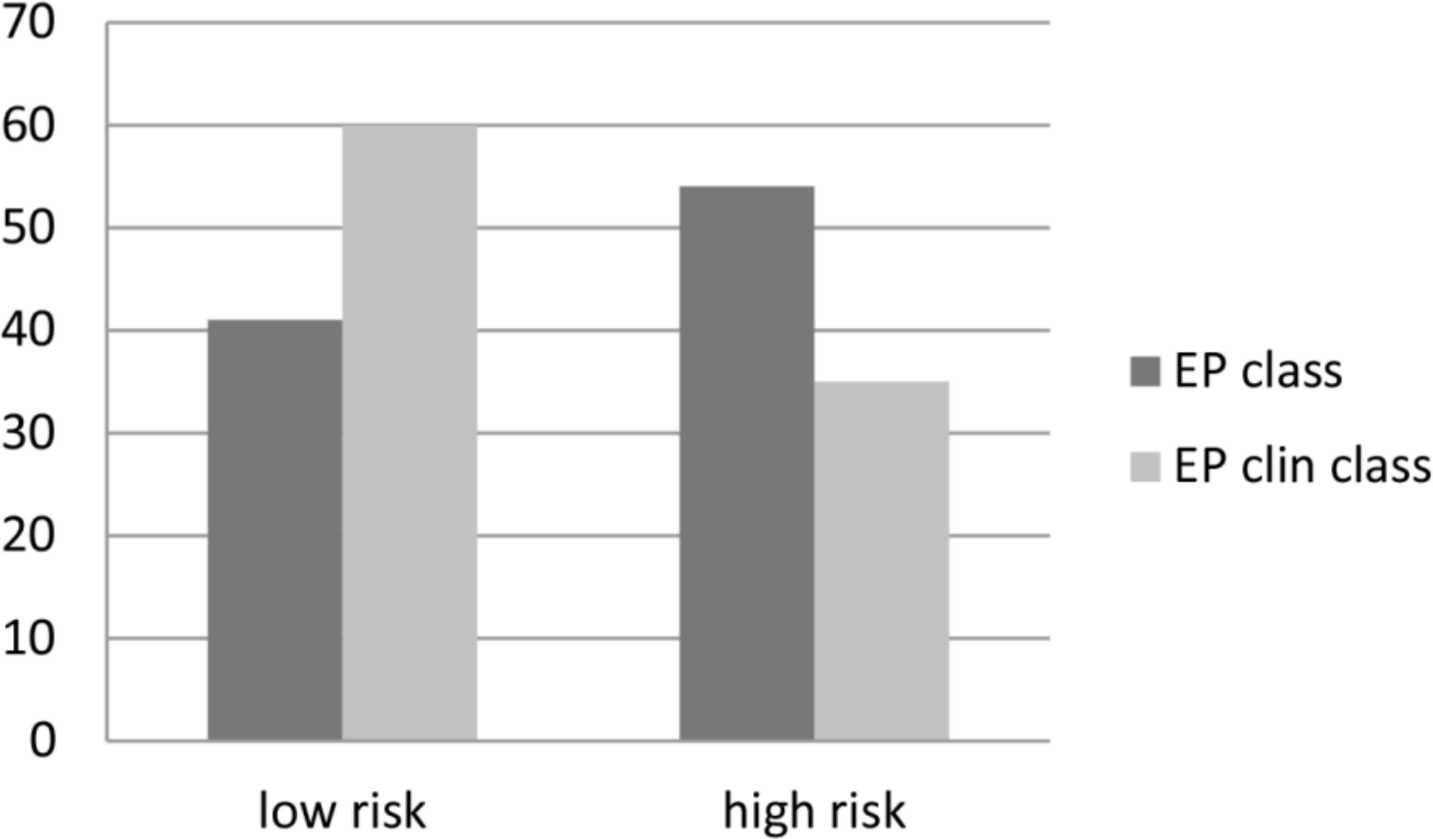
Risk distribution according to EP and EPclin. The X-axis indicates dichotomic risk based on each EP and EPclin classification. The Y-axis presents numbers of patients. Some patients have different results in EP assays according to EP and EPclin.

Regarding the molecular EP score, samples from 41 patients (43.2%) showed a low-risk and 54 patients (56.8%) showed a high-risk gene profile. After integration of clinicopathologic factors (tumor size and nodal status), the combined clinical and molecular score (EPclin) indicated low risk in 60 (63.2%) and high risk in 35 (36.8%) patients (Fig 2).

EPclin-based estimated median 10-year risk for metastases with endocrine therapy alone was 7% for the whole cohort. The estimated median risk was 6% for the EPclin-low group and 16% for the EPclin- high group.

### Comparison of EndoPredict with standard clinical parameters

**Fig 3:**
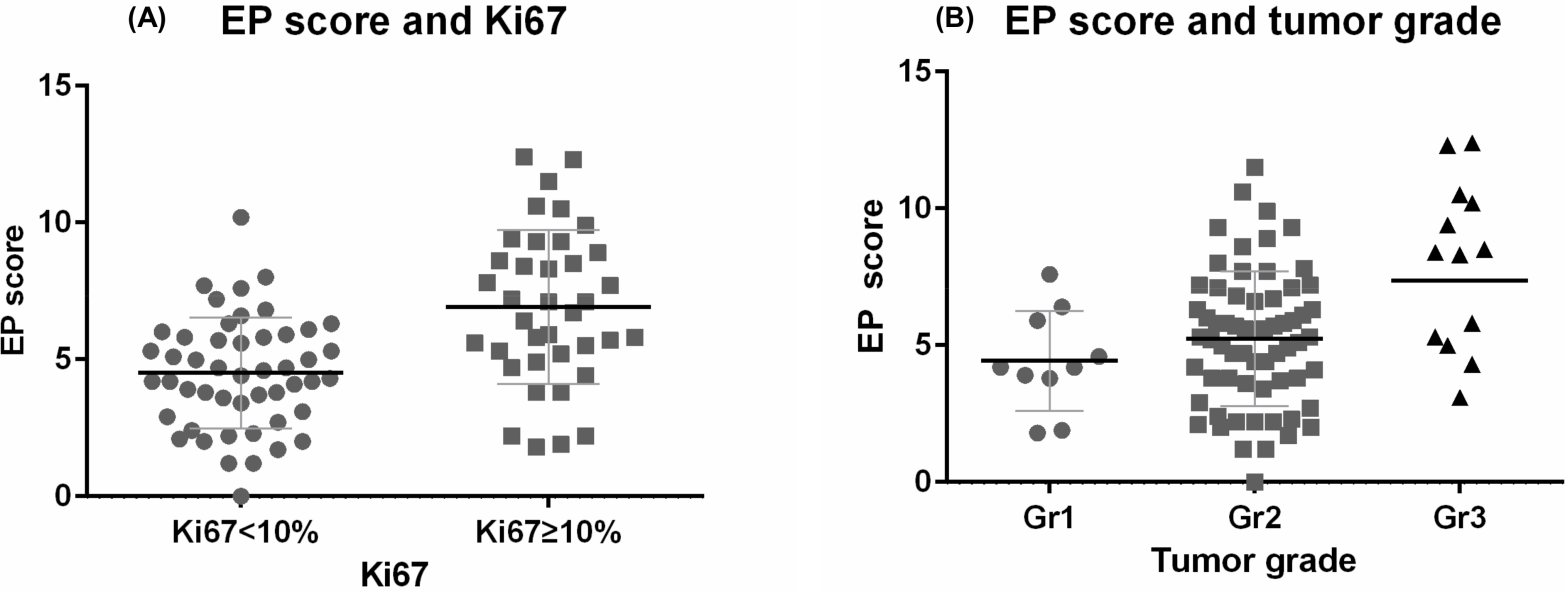
Distribution of the EP score. The cut-off point of Ki-67 was defined according to median point in our study population. The solid line revealed the median level of EP score in each subgroup. The EP score showed positive correlation to Ki-67 level and tumor grade. (A) In relation to Ki-67. (B) In relation to tumor grade.

The median EP score increased from 4.2 to 5.3 and 8.5 for tumors assigned G1, G2 and G3 grades. In addition, the EP score had a higher median value of 6.9 in tumors with a high Ki-67 index, compared to 4.5 in lingering proliferative tumors. Fig 3 provides an overview of the distribution of the molecular risk score EP depending on Ki-67 index (A) and tumor grade (B)

### Impact of WFO recommendations on changes in therapeutic decisions

**Fig 4:**
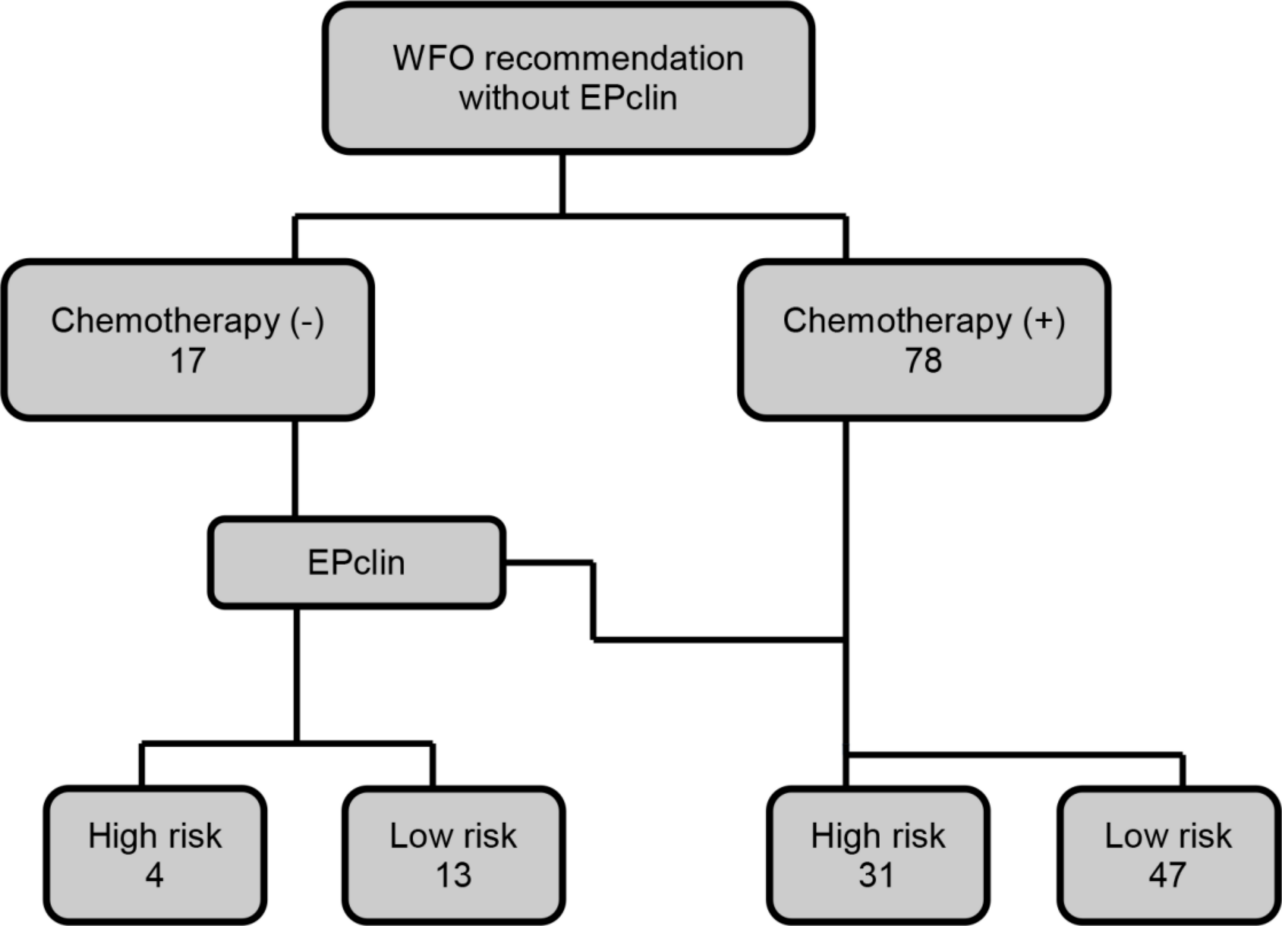
WFO assessment without EPclin in whole patients. In the WFO alone assessments, chemotherapy was recommended rather than endocrine therapy only.

**Fig 5:**
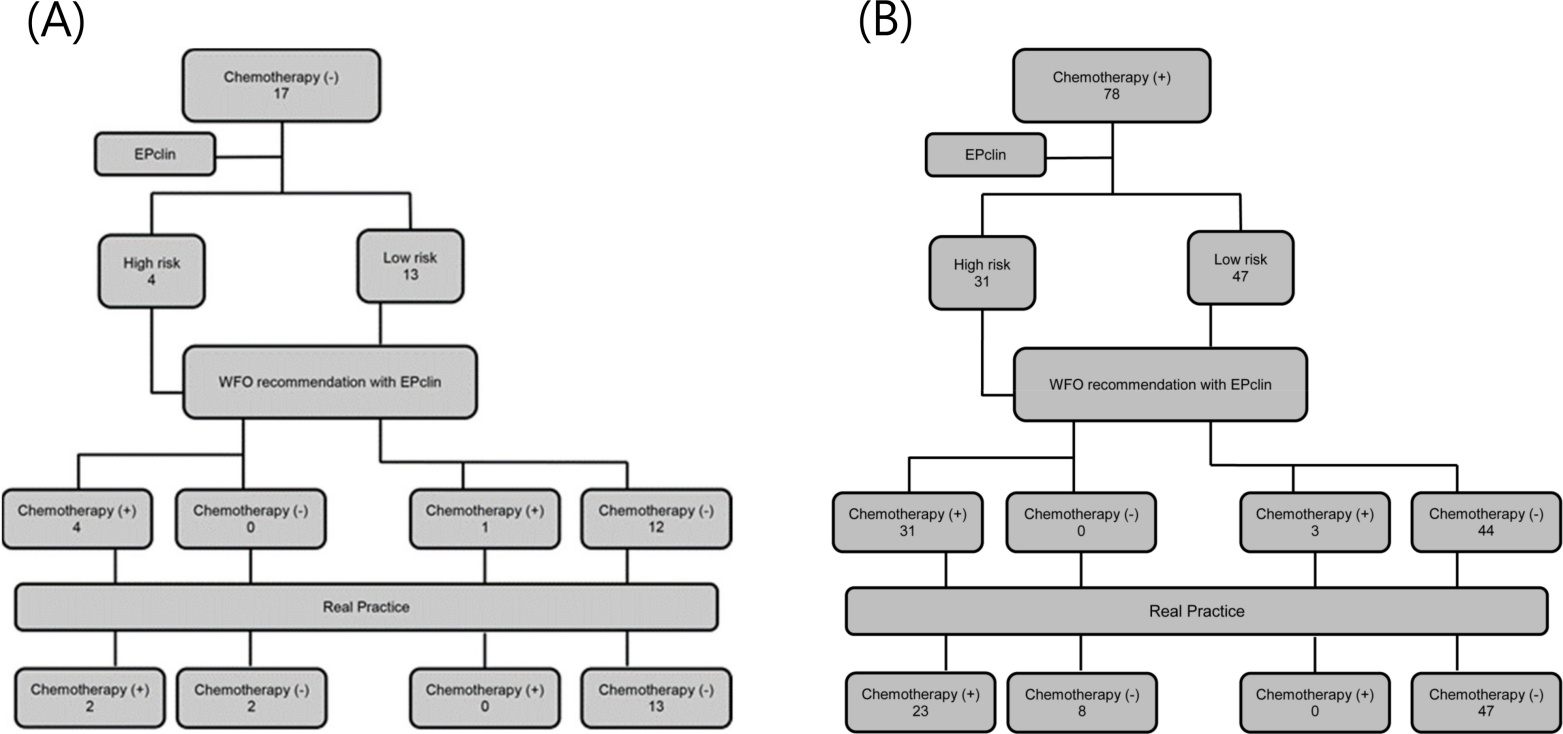
The change of WFO recommendations on therapeutic decisions by addition of EPclin results. WFO recommendations vary with or without EPclin. Based on results obtained with the WFO-EPclin combination, recommendations for chemotherapy were reduced substantially. (A) Subgroup of ‘No chemotherapy’ according to WFO recommendations. (B) Subgroup of ‘Add chemotherapy’ according to WFO recommendations.

WFO results were assessed both with and without EPclin. In the WFO alone assessments, chemotherapy was recommended rather than endocrine therapy only (Fig 4). Patients were categorized into two groups, specifically, ‘No chemotherapy’ and ‘Add chemotherapy to endocrine therapy’, as shown in Fig 5. Based on results obtained with the WFO-EPclin combination, recommendations for chemotherapy were reduced from 78 to 23 patients. Chemotherapy was indicated in 57.9% patients with WFO, while in real clinical practice, only 23.2% patients received chemotherapy. Chemotherapy was added by WFO in cases with high EPclin scores. Compared with clinical practice, the sensitivity, specificity, positive predictive and negative predictive values of WFO without EPclin were 92%, 21.4%, 29.5%, and 88.2%, respectively. Tumor size was the most statistically significant factor according to WFO without EPclin for recommending addition of chemotherapy (OR 6.58, 95% CI 1.93–22.50, *p*<0.001). On the other hand, data obtained using WFO combined with EPclin showed good clinical applicability. The sensitivity, specificity, positive predictive and negative predictive values of WFO with EPclin were 100%, 80%, 61% and 100%, respectively. In real clinical practice, nodal positivity was the only significant factor for inclusion of chemotherapy in all cases (OR 21.61, 95% CI 2.65–175.93, *p*<0.001).

## Discussion

Owing to better understanding of the utility of biologic markers, such as ER, PR, and HER2, in treatment guidance, tumor biology has become a surrogate of prognosis in BC. Different therapeutic strategies based on biologic subtype have gained prominence in the clinic in recent years. Besides the predictive power of intrinsic breast cancer phenotypes (such as luminal, HER2 and basal), anatomic staging systems are associated with outcomes, offering predictive synergy. This creates a dilemma that has a particular impact on prognosis in cases of discordance between staging and biological data. In an earlier study, Park and co-workers suggested that patients with node-positive luminal A breast cancer may not benefit from systemic chemotherapy [25]. According to the group, multigene assays should provide further predictive power to identify non-responders to chemotherapy and may be effectively employed to avoid chemotherapy in a large number of patients. However, under the anatomic-based staging system, node-positive BC cases are regarded as having physical lymphatic spread and recommended for chemotherapy, regardless of biology.

Following efforts to incorporate biologic factors in the American Joint Committee for Cancer (AJCC) staging, the 8^th^ edition prognostic stage groups also take into consideration multigene panel testing [26, 27]. Although the majority use Oncotype Dx, changes in stage can be up or downgraded by combining genomic profiling results with conventional TNM staging. For example, some groups anatomically classified as T2N0M0 (stage 2A) were downgraded to stage 1 owing to low RS in Oncotype Dx, clearly indicating that staging complexity is increased using the multigene panel assay. To determine the suitability of chemotherapy for a group of interest, artificial intelligence programs are required to provide reliable answers to clinicians and the general population.

In our study, Watson recommended the addition of chemotherapy in most cases when genomic data were not incorporated into the calculation. Chemotherapy was not recommended for 17 out of 95 patients. Differences in chemotherapy indications were related to tumor size and staging. Interestingly, upon integration of EPclin results with WFO and re-assessment of the group for which chemotherapy was not suggested, 5 out of 17 patients were recommended chemotherapy. These 5 patients included 4 with high-risk and 1 with low-risk EPclin scores. The patient classified as low-risk had node-positive BC. In contrast, chemotherapy was not recommended for 44 of 78 patients, among which 31 were grouped as high-risk and 47 as low-risk according to EPclin data. Despite being categorized as low risk, chemotherapy was recommended for 3 patients, since they all had node-positive BC. A higher proportion of patients did not receive chemotherapy in real practice. However, high-risk tumor was detected in 10 of these cases. Clinicians' discretion, clinical decisions, low levels of Ki-67 and patient opinion were associated with these findings. In the clinic, the Ki-67 level is regarded as an important prognostic factor. However, Ki-67 index as a single marker of proliferation was not considered for addition to staging due to its known lack of reproducibility as well as discrepancies in the optimal cutoff point between different laboratories. Our data clearly indicate that Watson combined with EPclin has clinical utility for consideration of chemotherapy, with sensitivity, specificity, positive predictive and negative predictive values of 100%, 80%, 61% and 100%, respectively. Relatively low clinical utility of WFO without genomic data is expected. However, the combination of WFO and genomic risk assay is of considerable utility, and yields similar therapeutic choices to those recommended by clinicians. Therefore, the IBM formula should consider inclusion of genomic assessment as essential rather than optional.

Our study had several limitations. First, several unknown confounding factors may affect the results due to its retrospective design. Secondly, a relatively small number of patients were enrolled in our study group. Lastly, the use of different genomic assessment tools could lead to calculation errors in the IBM formula. The IBM formula originally uses Oncotype Dx data with three risk types (classified as low, intermediate, and high risk groups). However, EPclin generates low and high dichotomous classifications. The predefined cut-off values for diagnostic decisions corresponding to 10% distant recurrence rate at 10 years are applied to stratify patients into EPclin low-risk (<3.3) and EPclin high-risk (≥3.3) groups [17]. Based on RS from Oncotype Dx, cut-off values of 18 and 31 in the NASBP B-14 trial cohort corresponded to ~11% and 20% 10-year risk of distant recurrence. Buus et al. demonstrated concordance between EPclin and RS categorization of risk for most cases in their study population [28]. The group directly compared recurrence rates in these categories with low-/high-risk categories of EP and EPclin and pooled the RS intermediate-and high-risk groups to create an RS non-low-risk group. The EPclin low-risk classification was further confirmed based on the similar number of patients categorized as low risk using RS coupled with a substantially lower 10-year recurrence rate. Kaplan-Meier plots for 10-year distant recurrence were generated according to EP, EPclin and RS scores in all patients stratified by EPclin low/high vs RS low/non-low groups. Each group showed equality using the log-rank test and patients in each group were largely distinguished according to low/high risk [28]. Other reports have demonstrated a similar concordance between RS and EPclin scores [29]. We separated the study groups into low vs high (non-low in RS) risk with EPclin, and entered the EPclin results into WFO instead of RS. While the discordance in results from EPclin and Oncotype Dx was regarded as insignificant, the bias from different genomic tools should be adjusted within the IBM formula. With the addition of various genomic datasets to the IBM Watson formula, more precise prediction should be possible in the future.

## Conclusions

In terms of making informed therapeutic decisions and selecting chemotherapy for treatment of ER-positive, HER2-negative early BC, WFO may deliver significant benefits for clinicians.

1. In ER-positive, HER2-negative early BC cases with low clinicopathologic or genomic risk, chemotherapy is not recommended. Risk stratification by WFO may be not be sufficient for indication of the appropriate therapy.
2. Artificial Intelligence (AI) should be intensively developed and improved to maintain consistency with real diagnostic reasoning performed by human clinicians. Genomic risk assessment may play a positive role in elevating the concordance rate between clinical practice and WFO therapeutic options.
3. We believe that the differences between real practice and Watson do not indicate the superiority of one form of diagnosis (computed versus human) over the other. However, clinicians are ultimately responsible for treatment. AI delivers emerging genomic information but requires further development for effective implementation in the unfolding genomic revolution.
